# The impact of electronic cigarettes on the outcomes of total joint arthroplasty

**DOI:** 10.1007/s00402-024-05565-2

**Published:** 2024-11-06

**Authors:** Hussain Zaidi, John Stammers, Ahmed Hafez, Philip Mitchell, Sulaiman Alazzawi, Alexandros Maris, Alexander Maslaris

**Affiliations:** 1 Department of Trauma and Orthopaedic Surgery, Complex Arthroplasty Unit, St Georges University Hospital NHS Trust, London, UK; 2 University College London, Royal Free Hospital, London, UK; 3 Nuffield Orthopaedic Centre, Hip and knee team, Oxford University Hospital NHS Trust, Windmill Rd, Oxford, OX3 7LD UK; 4 Department of Orthopaedics, Waldkliniken Eisenberg, Chair of the Jena University Hospital, Klosterlausnitzer Str. 81, 07607 Eisenberg, Germany

**Keywords:** Nicotine, Electronic cigarettes, Vape, Total knee arthroplasty, TKA, Total hip arthroplasty THA, Complications, Infection, PJI, Wound healing, Aseptic loosening

## Abstract

**Background:**

Cigarette smoking is known to result in poorer outcomes for patients undergoing total joint arthroplasty. Smoking tobacco cigarettes in the perioperative period is associated with higher analgesia usage, increased mortality, poorer healing, and an increased risk of medical complications. As such, many surgeons advise their patients not to smoke in the perioperative period. Electronic cigarettes are emerging as a popular alternative for usage by patients who would otherwise continue to smoke traditional cigarettes. Importantly, there has been limited investigation into the impact of electronic cigarette usage on the outcomes of total joint arthroplasty. This review investigates the potential detrimental effects caused by the usage of electronic cigarettes on the outcomes of total joint arthroplasty.

**Methods:**

A systematic review was carried out in accordance with the PRISMA Guidelines. We have drawn from studies that investigated the impact of the constituents of E-cigarette vapour on bone health, wound healing, the immune system and the direct impact of electronic cigarette usage on surgical outcomes.

**Results:**

Electronic cigarettes release nicotine in an inconsistent manner, resulting in many negative consequences for bone health. Furthermore, they depress the immune system, impair wound healing and may result in longer hospital stays.

**Conclusions:**

Electronic cigarette usage should be monitored in the perioperative period to reduce the risk of complication. There is a pressing need for more comprehensive research in this area to fully understand the implications of EC usage on the outcomes of total joint arthroplasty.

## Introduction

Traditional cigarette smoking is known to impair post-operative wound healing and increase the likelihood of both superficial and deep wound infections in patients who have undergone total joint arthroplasty [1]. Smoking cessation four weeks prior to procedure is known to reduce the occurrence of such complications, making it a modifiable risk factor [2]. Electronic cigarettes [EC] are often recommended to be used as alternatives to traditional cigarettes; guidance from Public Health England suggests that E-cigarettes are 95% safer than cigarettes, exacerbating the public perception of negligible risk [3]. However, there is an increasing volume of research detailing the harmful effects of vaping which may poorly influence the outcomes of orthopaedic surgery. [4–23]. This article discusses the impact of electronic cigarette usage, commonly referred to as ’vaping’, on the immune system, bone physiology, anaesthesia and other surgical complications in patients undergoing total joint arthroplasty.

Categorising outcomes in total joint arthroplasty (TJA) is a multifactorial process, encompassing both patient and clinician-decided criteria. A good outcome is clinically characterised by the successful alleviation of pain and restoration of joint function allowing for improved mobility and activity. Achieving this involves the seamless integration of the prosthetic components within the patient’s bone and soft tissues, minimising the risk of complications such as infection, dislocation, or prosthetic failure. From a patient’s perspective, good outcomes are often measured in terms of an enhanced quality of life, such as increased activity levels and reduced reliance on pain medications. Long-term success is defined by the durability of the implant and the lack of the need for a revision. Collectively, these factors constitute the benchmarks for evaluating a successful total joint arthroplasty. The aim of the study was to conduct a thorough literature review into the effects of electronic cigarettes on the outcomes of total joint arthroplasty, aiming to identify the potential risks and limitations of electronic cigarette usage during the perioperative stage.

**Fig. 1:**
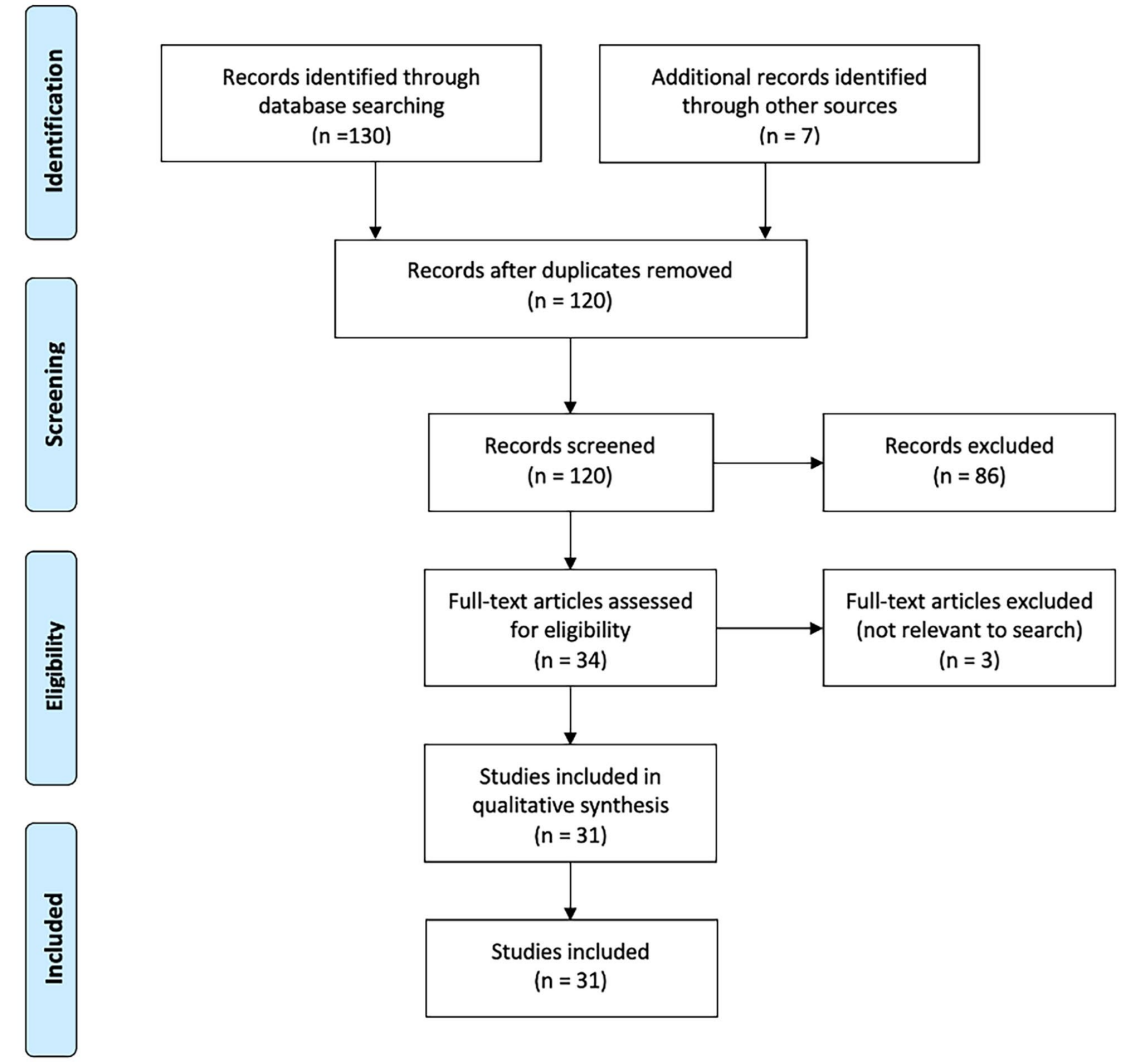
PRISMA flowchart

**Fig. 2:**
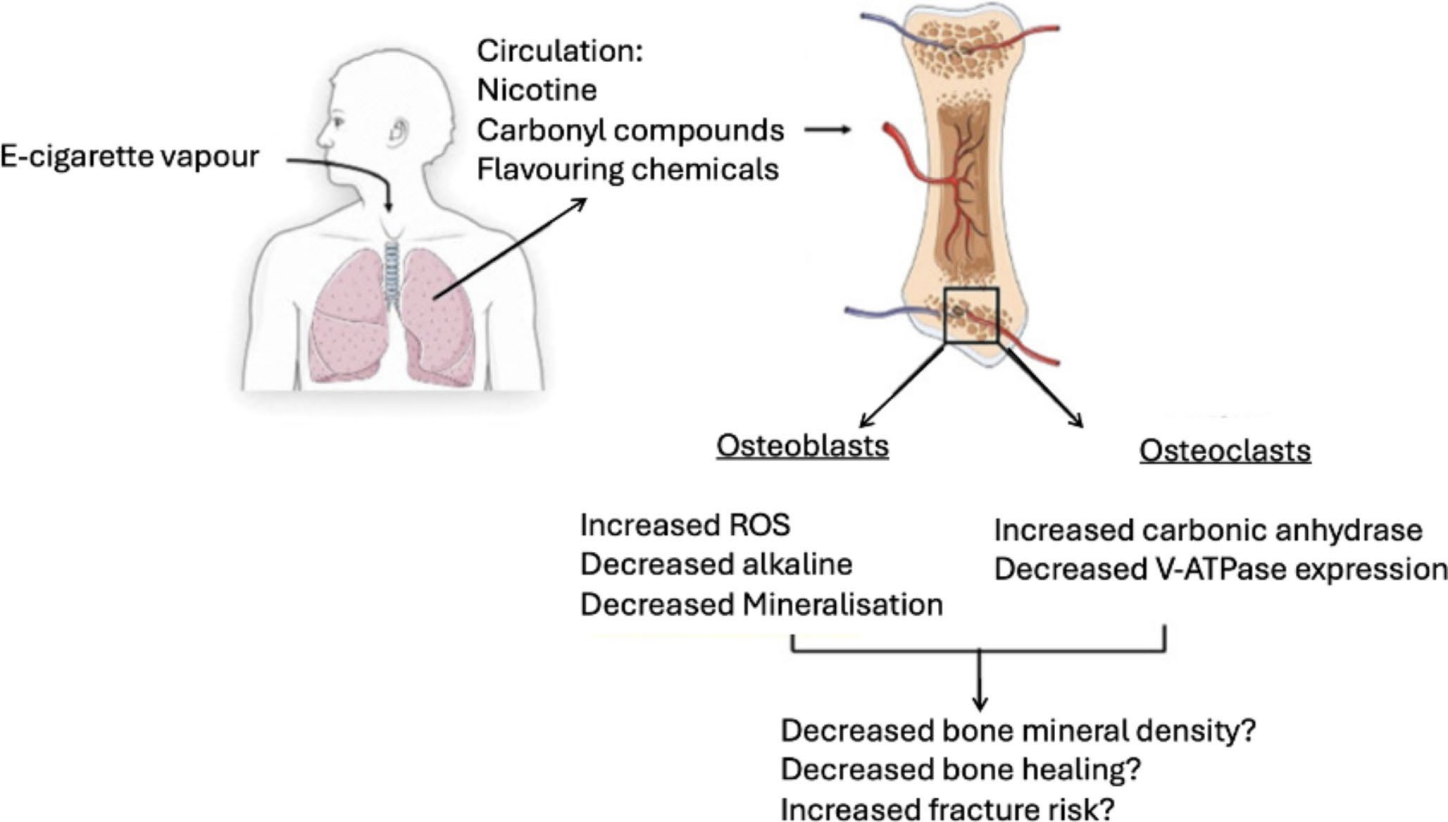
A potential mechanism to highlight how E-cigarette usage may impact bone. Adapted from Nicholson et al. [11]

**Table 1:** A comparison of the outcomes of 21341 patients with recorded smoking status who underwent primary total joint arthroplasty. Adapted from Bieganowski et al. [9]

Outcomes					
	Zero exposure (*n* = 11,459)	Tobacco only (*n* = 8857)	Both tobacco and EC (*n* = 664)	EC only (*n* = 44)	*P* value
Surgical time (± SD), min	97.84 ± 31.00	97.65 ± 30.39	103.04 ± 31.71	110.36 ± 41.59	< 0.001
Length of stay (± SD), days	2.09 ± 1.76	2.09 ± 1.61	2.19 ± 1.49	2.34 ± 3.73	0.01
Discharge disposition					0.648
Home	88.20%	89.00%	88.30%	84.10%	
Facility	11.80%	11.00%	11.70%	15.90%	
Readmission rate	3.20%	4.20%	4.50%	9.10%	0.001
Revision rate	2.40%	3.00%	2.60%	4.50%	0.06
Zero exposure = No exposure to either EC or tobacco					

## Methods

The literature reviewed in this paper was gathered from a variety of sources; Pubmed, MEDLINE, Cochrane Library, and Google Scholar. A search was carried out in April 2024, with search terms “electronic cigarettes or e-cigarette or e cigarette or vape* or vaping or electronic nicotine).mp. AND ortho* or surgery).mp.”. Further searches for additional material were carried out on the Pubmed and Cochrane databases and Google Scholar. Discrepancies were ameliorated by mutual discussion.

During our search, it became apparent that there is a dearth of literature investigating the impact of the usage of electronic cigarettes on the outcomes of total joint arthroplasty. There is a lack of human clinical studies, and a general paucity of information and evidence regarding electronic cigarettes and orthopaedic surgery; this area should be a potential target for future research. In some situations, we extrapolated information from basic science papers and literature covering other surgical procedures, where the underlying physiology remains the same.

### Inclusion criteria

We included all clinical studies, randomized and non-randomized clinical trials, multicenter studies, case reports, basic science papers, reviews, systematic reviews, and meta-analysis reporting on the outcomes of surgery, bone health, and the impact of electronic cigarettes on wound healing and the immune system. There were no limitations chosen for publication date and study population. Studies not published in the English language were excluded.

## Results

### On the epidemiology of electronic cigarettes

Electronic cigarettes aim to mimic the experience provided by conventional cigarette smoking. They vaporise a solution usually composed of water, propylene glycol, glycerine and nicotine. Because ECs do not contain tobacco, and are combustion free, they are commonly assumed to be safer than conventional cigarette smoking. Marketing campaigns suggest that ECs are a healthier alternative to traditional tobacco cigarettes. A range of available flavours and, in comparison to traditional tobacco cigarettes, reduced marketing limitations, have helped increase the prevalence of these devices. Whilst initially positioned primarily to aid smoking cessation, electronic cigarettes have become a gateway to tobacco and marijuana usage, including for adolescents. It is estimated that there are 4.5 million users of electronic cigarettes, and 6.4 million traditional smokers in the United Kingdom. In 2022, vaping prevalence was found to be 8.6% in children aged 11–18 compared to 4% in 2021 [24]. There is also increasing harm to the environment, with 52.8% of users reporting usage of disposable EC products in 2022; compared to just 5.3% in 2020. Smoking prevalence in the same age group was found to be 6%.

Electronic cigarette usage, commonly referred to as “vaping”, has been found to be associated with increased incidence of a multitude of harmful conditions. Some of the chemicals in e-liquid aerosol have been linked to lung damage, including bronchiolitis obliterans. ECs have also been found to negatively affect cardiovascular health, potentially elevating blood pressure and heart rate. Long term usage may also lead to atherosclerosis [25]. Furthermore, there is growing evidence that e-cigarette usage negatively impacts mental health, with a greater effect on adolescents. E-cigarettes may also result in increased aggressive and impulsive behaviour, poorer sleep quality, attention deficits, impaired memory and cognition, and increased depression and suicide ideation [4].

### The effect of ECs on bone health, and the resulting impact on the outcomes of THA

Cigarette smoking is known to reduce bone mineral density, increase risk of fracture and negatively impact fracture healing [1]. Cigarette smoking also increases the likelihood of post-procedural infection and aseptic loosening following total hip arthroplasty [5]. But what about E-cigarettes?

It is hard to draw a definitive conclusion from the limited research. There are no randomised controlled trials investigating the impact of electronic cigarette usage on orthopaedic outcomes. Risk factors for impaired bone healing include age, increased alcohol consumption, nutritional deficiency, pre-existing medical conditions and tobacco smoking. Although ECs reduce user exposure to some of the toxins found in tobacco cigarettes, they contain many potentially harmful substances in the dilutants used to contain nicotine [6]. EC vapour contains nicotine, flavouring chemicals and other reactive aldehydes; particularly bad for highly vascularised tissue such as bone. We shall independently investigate the effect that each of these constituents has on bone.

The impact of nicotine overexposure on bone physiology has raised concerns. Nicholson et al. suggested that the raised and inconsistent levels of nicotine found within e-cigarettes could impair the function and viability of both osteoclasts and osteoblasts [11]. The presence of nicotine has been reported to upregulate expression of nicotinic acetylcholine receptor subunits in both primary human osteoblasts and human bone tissue. Walker et al. described the “bimodal effect” nicotine may have on human bone cells such as primary human osteoblasts, where higher concentrations of nicotine triggered cell death [26]. Similarly, Marinucci et al. reported on osteoblast apoptosis induced by nicotine; resulting from the accumulation of reactive oxygen species [27]. Low doses of nicotine stimulate proliferation whilst higher doses significantly reduce proliferation and induce apoptosis.

Electronic cigarettes are known to deliver a highly unpredictable and variable nicotine content (of range 0 to 20 mg/mL); [28] not much better compared to traditional cigarettes (6.17 to 28.86 mg) or nicotine replacement therapy (5 to 52.5 mg) [8]. To further exacerbate this issue, E-cigarettes are often used as an additional device to aid smoking cessation alongside traditional tobacco aids, such as patches; EC usage in combination with other methods of nicotine delivery can result in inadvertently raised levels of serum nicotine [9]. Furthermore, there is evidence to suggest EC users increase nicotine consumption to achieve a similar dose to what they would have obtained from cigarettes, increasing the potential for nicotine-induced harm [10]. Therefore, the impact of nicotine on bone following the use of nicotine-containing E-cigarettes may be comparable to or greater than those of conventional cigarettes.

Chronic exposure of greater than six months to EC vapour free of flavourings and nicotine has been shown in vivo to have a substantial impact on bone architecture, with microfractures found in the femurs of mice [11, 12]. However, no effect on cortical bone strength, stiffness or hydroxyapatite content was observed. There are very few studies investigating the physiological effects of the flavouring chemicals found within ECs on bone directly.

From these studies, one can draw the conclusion that nicotine overexposure is highly detrimental to outcomes of total joint arthroplasty. There is a lack of content regulation, and poor-quality control of the flavouring compounds of EC fluids; nicotine is not delivered consistently, and the e-cigarette smoker is exposed to potentially higher levels of nicotine than they would receive from a traditional cigarette, elevating the risk of developing addiction.

### E-cigarettes depress the immune system, increasing risk of infection

Emerging evidence indicates EC usage negatively impacts both innate and acquired immunity. EC vapour has been found to increase production of reactive oxygen species, inflammatory cytokines and chemokines resulting in a prolonged inflammatory state, whilst the inhibition of phagocytosis suggests EC-use impairs bacterial clearance [13]. Keith and Bhatnagar [14] found EC usage induces extensive immune changes; different from those caused by traditional cigarettes. In healthy, smoke-naïve individuals, serum levels of reactive oxygen species and ICAM-1 have been shown to increase within two hours of exposure [15] whilst chronic EC users show raised plasma IgE levels [16]. The authors found EC users showed greater gene suppression than smokers of traditional cigarettes. Whilst the long-term implications of these results are currently unknown, the authors hypothesise that EC-induced reduction in chemokines at the epithelial level could dampen the recruitment and activation of innate immune cells to defend bacterial, viral, and fungal infection. Clapp et al. reported that some of the flavourings used in e-cigarettes may further blunt the immune response, beyond the damage caused by raised nicotine levels [17]. Furthermore, Page et al. found EC exposure decreased cutaneous blood flow; potentially impairing wound healing [18]. All of these factors may poorly affect the outcomes of recovery and wound healing post-total joint arthroplasty.

Flavouring chemicals are now known to have a plethora of harmful effects on the function of immune cells. Atomisation of flavouring chemicals, such as linalool, dipentene and citral, causes free radical production [19–29] independent of nicotine. EC vapour has also demonstrated cytotoxicity to endothelial cells [20]. The inhalation of EC vapour compromises vascular endothelial function and inhibits nitric oxide bioavailability; the result of this being decreased vasodilatation and increased platelet activation [30]. E-cigarettes and traditional cigarettes may have an equal detrimental effect on tissue oxygenation; Fracol et al. demonstrated similar levels of decreased subcutaneous blood flow with e-cigarette use. Through the release of catecholamines, Nicotine leads to increased hemodynamic instability under general anaesthesia and ECs may increase nicotine toxicity due to its increased and highly variable availability. Due to these factors’ negative effect on immune function, EC usage may lead to poorer infective outcomes. More research is needed in this area, specifically in relation to its effect on the perioperative outcomes of total joint arthroplasty.

### Vaping should be stopped in the perioperative period to decrease incidence of wound healing complications

Vaping has been documented to hold similar consequences to wound healing as cigarette smoking. Despite the lack of objective data, a 2023 literature review [31] found ECs should be treated similarly to tobacco cigarettes; therefore, vaping should be stopped in the perioperative period to reduce the risk of wound healing complications. As mentioned earlier, nicotine stimulates the release of adrenaline and other catecholamines, leading to peripheral vasoconstriction, reducing tissue perfusion [21]. Nicotine also increases production of thromboxane A2; a potent vasoconstrictor found within platelets [22]. As blood perfusion is essential for adequate healing to occur, the effects of the nicotine ingested through e-cigarette usage may lead to tissue ischemia, which can delay wound healing [23]. This review reported ECs significantly affected the circulatory system; causing increased oxidative stress and severe endothelial dysfunction. Vaping has also been found to predispose naïve patients to increased vascular resistance in the peripheral vascular bed. As mentioned earlier, the exacerbation of these factors predisposes e-cigarette users to more infections, delayed healing, and poorer wound complications.

### TJA patients who have only vaped may have a longer surgical times and length of stay

In a 2023 retrospective study, Bieganowski et al. investigated the trends in outcome of vaping in primary total joint arthroplasty (TJA) patients, in the only clinical study directly investigating the impact of vaping on TJA outcomes [9]. They compared the vaping habits of patients undergoing routine physical examination (RPE) to those who underwent TJA procedures at the same centre. The authors found documented EC usage to be uncommon, with only an average of 3% of patients being documented EC users. Out of those who had ever vaped, those undergoing a TJA were found more likely to be former vapers compared to those undergoing only an RPE. Patients were subcategorised; those that had never vaped (NV), former vape (FV) users or those that were currently vaping (CV). To allow for further analysis, TJA patients were grouped into further categories; whether they had no exposure to tobacco or vaping (were smoke naïve), tobacco exposure only, both tobacco and vaping or ECs only (VO). Out of the 21,024 patients, 11,459 had used neither tobacco nor ECs, 8857 only used tobacco products, 554 used both tobacco and ECs and only 44 patients solely used ECs (VO group). The authors found that reporting on the usage of electronic cigarettes was missing from 12.8% of TJA patients. After controlling for demographic differences, those who vaped only spent a significantly longer time in theatre (110.36 ± 41.59 min; *P* < 0.001) and had a greater length of stay (2.34 ± 3.73 days; *P* = 0.01) when compared with all other cohorts. Patients who only vaped also had significantly higher rates of hospital readmission (9.1%, *P* = 0.001), higher than those who smoked traditional cigarettes. In this study, significant differences were observed in surgical time, length of stay and readmission rates for TJA patients who only smoked to e-cigarettes.

However, there are some issues with this paper which hinder our ability to draw a definitive conclusion. Just were 44 patients in the VO group, comprising only 0.21% of the study population; the proportionally smaller sample size of this group limits our ability to draw direct comparisons. We are further limited by most EC users also using tobacco. Other than stating the previously discussed physiological impact of EC vapour constituents of bone physiology, the authors of this paper do not give any indication as to why the VO group had significantly higher mean surgical time and rates of readmission. These differences may also be due other factors not controlled for in the analysis, such as existing comorbidities. Factors such as more muscular, complex anatomy have not been stated as being controlled for. Further research is needed to resolve these issues; a larger, controlled study with a greater population of patients who only vape.

## Discussion

The emerging research suggests that electronic cigarettes have a complex and multifaceted impact upon bone health, the immune system and wound healing, resulting in the potential for poorer outcomes for patients undergoing total joint arthroplasty. This study is the first to comprehensively report on the impact of electronic cigarette usage on patients undergoing total joint arthroplasty, with the aim of raising awareness of the impact such devices on outcomes. The presence of nicotine, along with other constituents of EC vapour such as flavouring chemicals and reactive aldehydes, has been implicated in various adverse effects on wound healing, bone physiology and to the immune system. The impact of ECs on the immune system, including alterations in immune cell recruitment and activation raises concerns regarding their usage on post-operative infection risk and wound healing. Furthermore, recent data highlights the potential for increased time in theatre, and longer lengths of stay in hospital in patients who exclusively vape [17].

First patented in China in 2003, Electronic cigarettes are relatively novel; as a result, there is a dearth of literature investigating the long-term effects of EC usage [32]. The quality of evidence found in many studies is marred by poor sample sizes; therefore, their statistical rigor should be questioned. During my research, we identified issues relating to the way patients are identified. This should be rectified for future research. There are problems regarding the classification of patients who vape. There are distinctions to be made which are necessary but often neglected; between the category of patients who vape; between patients who have only ever utilised e-cigarettes and those who previously smoked traditional cigarettes and other products and now utilise e-cigarettes. There are different types of e-cigarettes, and the type used by a patient is often not recorded; this is a problem as the e-liquid contents can differ, including in levels of nicotine content and the presence of substances such as cannabis. Additionally, the period a patient has vaped for is often not noted, allowing for further differences within patient population. Future prospective studies should ensure that these factors are adequately recorded.

## Conclusion

Electronic cigarettes appear to have a detrimental effect on the outcomes of total joint arthroplasty, potentially resulting in longer operative times and post-operative length of stay. We should therefore be wary of recommending the usage of ECs as an alternative to traditional cigarettes in the perioperative period. Electronic cigarette usage should be monitored in the perioperative period to reduce the risk of complication. EC vapour is linked to increased cytotoxicity, inflammation and decreased anti-microbial efficacy, potentially increasing perioperative risk for patients undergoing total joint arthroplasty. Additionally, EC usage is detrimental to wound healing in a similar manner to smoking. Furthermore, the nicotine content of inhaled vapour has many detrimental effects which are exacerbated by characteristic inconsistent dosage of electronic cigarettes. The lack of published evidence has led to inconsistencies in whether surgeons recommend for patients to utilise E-cigarettes in the perioperative period as an alternative to traditional cigarettes. More research is needed; a prospective, propensity matched study investigating both the short term and long-term effects of electronic cigarettes on a large group of patients undergoing total joint arthroplasty. Until then, we argue that one should be wary of recommending the usage of e-cigarettes in the perioperative period. More research specific to the outcomes of total joint arthroplasty is needed; prospective clinical trials are needed to definitively evaluate the perioperative risks. Considering the above findings, it is apparent that vaping is not a risk-free alternative to traditional smoking for patients awaiting total joint arthroplasty.

## Data Availability

The data that support the findings of this study are available from the authors upon reasonable request.
